# Computational toolbox for the analysis of protein–glycan interactions

**DOI:** 10.3762/bjoc.20.180

**Published:** 2024-08-22

**Authors:** Ferran Nieto-Fabregat, Maria Pia Lenza, Angela Marseglia, Cristina Di Carluccio, Antonio Molinaro, Alba Silipo, Roberta Marchetti

**Affiliations:** 1 Department of Chemical Sciences, University of Naples Federico II, Via Cinthia 4, 80126, Italy

**Keywords:** computational tools, glycan–protein interactions, MD, molecular recognition

## Abstract

Protein–glycan interactions play pivotal roles in numerous biological processes, ranging from cellular recognition to immune response modulation. Understanding the intricate details of these interactions is crucial for deciphering the molecular mechanisms underlying various physiological and pathological conditions. Computational techniques have emerged as powerful tools that can help in drawing, building and visualising complex biomolecules and provide insights into their dynamic behaviour at atomic and molecular levels. This review provides an overview of the main computational tools useful for studying biomolecular systems, particularly glycans, both in free state and in complex with proteins, also with reference to the principles, methodologies, and applications of all-atom molecular dynamics simulations. Herein, we focused on the programs that are generally employed for preparing protein and glycan input files to execute molecular dynamics simulations and analyse the corresponding results. The presented computational toolbox represents a valuable resource for researchers studying protein–glycan interactions and incorporates advanced computational methods for building, visualising and predicting protein/glycan structures, modelling protein–ligand complexes, and analyse MD outcomes. Moreover, selected case studies have been reported to highlight the importance of computational tools in studying protein–glycan systems, revealing the capability of these tools to provide valuable insights into the binding kinetics, energetics, and structural determinants that govern specific molecular interactions.

## Review

### Introduction

Carbohydrates also referred to as saccharides, sugars, or glycans, constitute one of the main building blocks of biomolecules, alongside lipids, proteins, and nucleic acids. In humans and animals, they form the so-called glycocalyx, a protecting sugar coat decorating the cell surface and modulating a myriad of cell–cell interactions [1]. It is composed of branched or elongated glycan chains covalently linked to proteins or lipids, hereby constituting glycoproteins or glycolipids, respectively. Recently, glycan structures exposed on the cellular membrane have also been found to be associated with tRNA [2]. In other species, such as prokaryotes, plants or fungi, glycoconjugates comprise the cell wall, playing critical metabolic, structural and physical functions [3].

**Figure 1:**
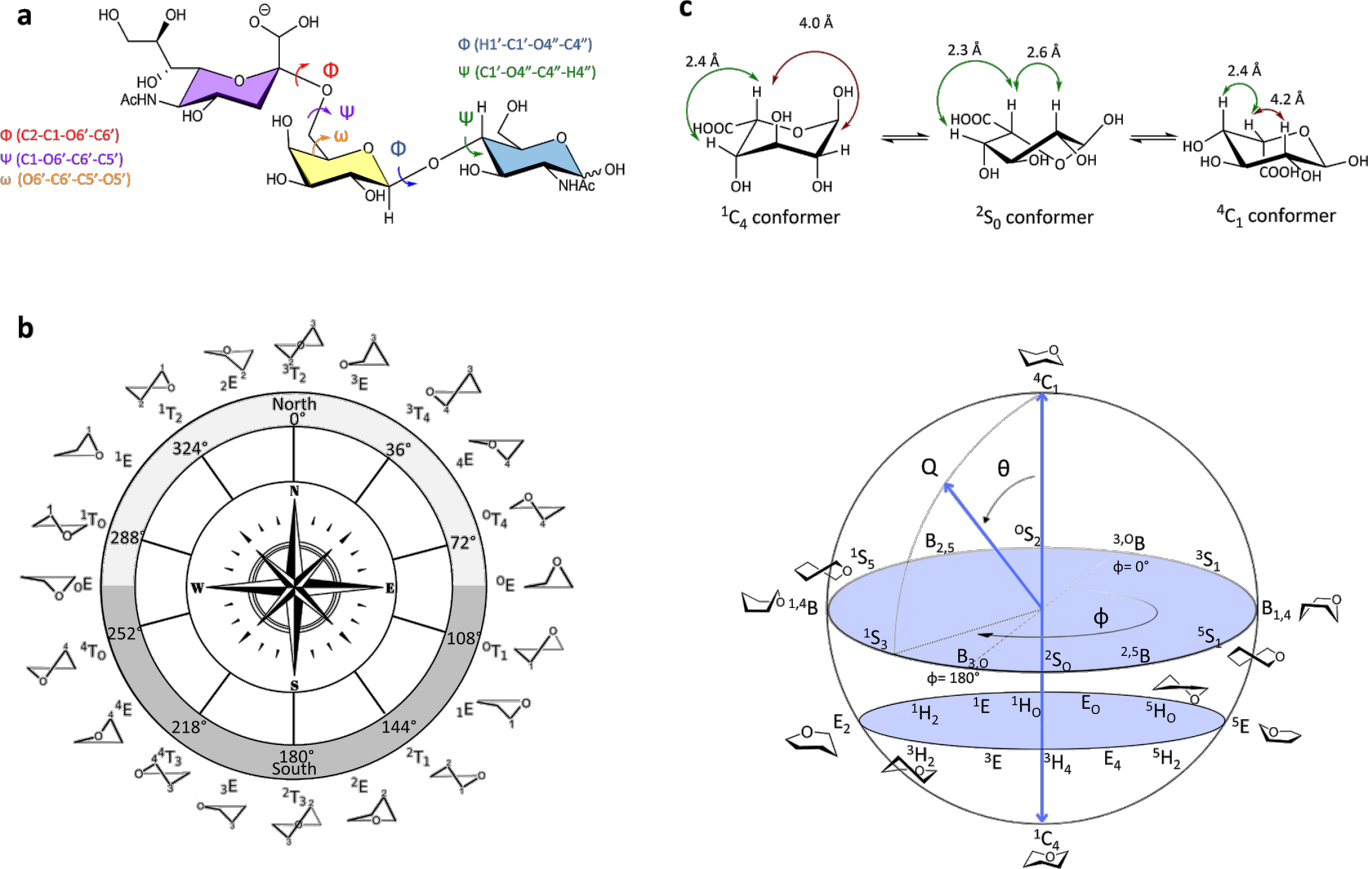
Carbohydrate conformational variability. a) Illustration of Φ, Ψ and ω dihedral angles for a representative trisaccharide coloured according to the symbol nomenclature for glycans (SNFG). b) On the left: Pseudo-rotational wheel depiction of five-membered ring structures showcasing envelope (E) and twist (T) conformations. On the right: Glove representation illustrating the puckering of six-membered pyranoside ring conformations. c) Equilibrium between the low-energy solution conformations of the iduronic acid. The exclusive (Nuclear Overhauser Effect) ^1^H–^1^H NOE contacts characteristic of each conformation, ^1^C_4_, ^4^C_1_ and ^2^S_0_, are also depicted.

Glycoscience encompasses the comprehensive study of glycans focusing on their structural, biosynthetic, biological and evolutionary aspects [4], thus playing a central role in the identification and characterisation of the glycome’ structure and function, and in unveiling its interaction with host proteins [5–6]. Notably, the complexity of the glycome far surpasses that of the genome, transcriptome, and proteome, not only due to the structural and conformational diversity of glycans, whose synthesis is not template driven, but also due to their dynamic nature [5–6]. Although mammalian glycans rely on a group of “only” 10 monosaccharide units, they can be assembled, in linear or branched chains, through different glycosidic linkages and diverse spatial orientations, which can also undergo modifications, such as methylation, sulfation, and phosphorylation, resulting in a plethora of different and particular structures [7–8]. Additionally, glycans can adopt a wide variety of different shapes; five-membered ring sugars can exhibit envelope and twist conformations usually represented on a pseudo-rotational wheel; while six-membered ring structures can adopt chair (C), boat (B), skew (S), and half-chair (H) conformations (Figure 1). Among them, chair’ shapes typically have the lowest energy and are thus preferred, except few cases in which different conformations can exist in a dynamic equilibrium, as for the iduronic acid that can adopt three low-energy solution conformations (Figure 1): ^1^C_4_, ^4^C_1_ (chair forms) and an additional skew-boat shape (^2^S_0_) [9]. The glycosidic torsion angles Φ (H_1_–C_1_–O_x_–C_x_) and Ψ (C_1_–O_x_–C_x_–H_x_) describe the relative orientation of two connected monosaccharide units; moreover, when dealing with monosaccharides containing an exocyclic hydroxymethyl group, such as in the case of 1-6 linked sugars, an additional torsion, namely ω (O_6_–C_6_–C_5_–O_5_), must be defined and three staggered conformers, denoted as gg/tg/gt (ω angles of −60°/180°/60°, respectively), should be considered (Figure 1).

Longer and branched glycans exhibit heightened structural dynamics, depending on the values adopted by the torsional angles around the glycosidic linkages [10].

**Figure 2:**
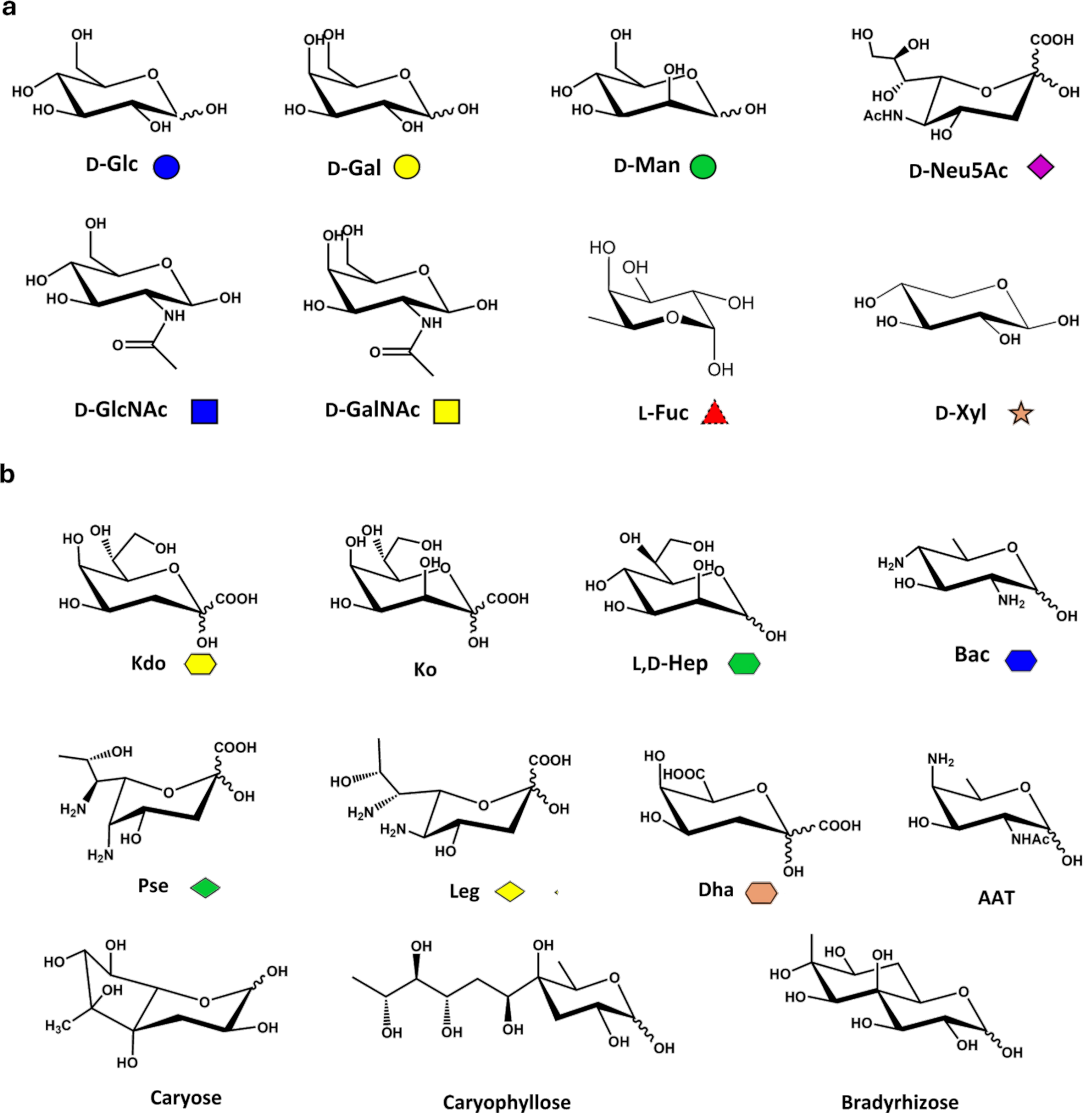
Monosaccharides diversity in eukaryotes and bacteria. a) Eukaryotic monosaccharides. b) Examples of some peculiar bacterial monosaccharides, including hexuronic acids, heptoses, or octulosonic acids. The SNFG symbol [11] of each monosaccharide is also reported (if any).

The high variability of linkages type, branching, stoichiometry, anomeric configuration (alpha and beta), and conformation contributes to the intricate nature of glycans. The complexity of the glycome is even higher in bacteria, which are able to use most of the mammalian sugar units to construct their glycoconjugates but, in addition, can also use a wide variety of particular, and potentially endless, monosaccharides that are instead not present in eukaryotes (Figure 2).

This huge diversity and complexity, especially in bacterial glycans, makes the structural and conformational analysis of glycans extremely difficult, posing a considerable challenge when employing conventional structural biology methods for glycan analysis [10,12]. Nevertheless, understanding the three-dimensional structure of glycans is crucial for comprehending their roles and biological activities and for correlating their structural features with their activity [3,13]. Given the plethora of remarkable biological roles played by complex glycans, this knowledge is essential for their potential applications in promoting health benefits for humans, animals and plants, including drug design [14–15], vaccine development [15–16] and numerous other possibilities in the field of carbohydrate chemistry and biology.

Notably, the regulation of the host immune response is often mediated by glycans, particularly through their recognition by a wide array of glycan-binding proteins (GBP) [17], which have a unique capability to specifically interact with endogenous and/or exogenous glycans [18–19]. Thus, disclosing the molecular basis of protein–glycan interactions has a unique potential to help modulate a myriad of complex biological events affecting the health and well-being of living organisms and the natural environment. Being key participants in the molecular dialogue, glycan binding proteins emerge as fascinating and critical components of molecular events that regulate life at its core. Their functions span from the catalysis of chemical processes [20–21], transporting and storing molecules [22], transducing and integrating information [23] providing structural and mechanical support [24], and generating movement [25], among other functions [26]. To fold and carry out their function properly, proteins often need post-translational modifications, including glycosylation, in which a carbohydrate chain is directly attached to a specific amino acid to generate glycoproteins and proteoglycans [27]. Based on the amino acid involved in the link with the carbohydrates chain, it is possible to classify different types of glycosylation: i) *N*-glycosylation, where a *N*-acetylglucosamine (Glc*N*Ac) is linked to the nitrogen atom of an asparagine side chain [28]; ii) *O*-glycosylation, where a Glc*N*Ac or *N*-acetylgalactosamine (Gal*N*Ac) is linked to the hydroxy group of a serine or threonine residue [29]; iii) *C*-glycosylation, where a mannose (Man) directly binds a tryptophan residue [30]; iv) the covalent attachment to core protein of glycosaminoglycans (GAGs), anchored to a Ser, or at lesser extent to Thr or Asn, forms proteoglycans. GAGs are complex negatively charged polysaccharides composed by disaccharide repeats of Glc*N*Ac or Gal*N*Ac combined with uronic acid (glucuronic or iduronic acid) or galactose residues, forming chains which can also be partially sulfated. GAGs family includes heparan sulphate (HS), dermatan sulphate (DS), chondroitin sulphate (CS), keratan sulphate (KS), and hyaluronic acid (HA) [31]. The extraordinary proteins versatility places them at the core of almost every biological event, including cell–cell communication and regulation of immune responses. In the majority of cases, these mechanisms are significantly influenced by the molecular interactions occurring between glycans and receptor proteins.

A well-known family of GBPs is constituted by the lectins, ubiquitous receptors that exhibit the ability to specifically recognise different carbohydrates through their well-defined binding pocket and they conserved three-dimensional structure similarities [32]. On the other hand, GAG-binding proteins, which are able to recognise carboxylic acid and sulphate groups along glycosaminoglycan chains using clusters of positively charged amino acids [33], also mediate a wide variety of cell–cell and cell–pathogen communication, controlling immune cell functions, and overseeing cellular trafficking [34]. Another class of GBP is represented by anti-carbohydrate antibodies, that are generally produced by the host organism for example against bacterial, fungal, and viral carbohydrates [35].

Given the wide variety of biological processes influenced by the protein–glycan interplay, an increasing attention has been focused in the last decades on the development of new techniques and technologies for the systematic analysis of complex glycans and the study of their interactions with proteins. A multidisciplinary approach, spanning from wet laboratory experiments and biophysical techniques to bioinformatics methods is needed to deeply investigate the multifaceted aspects of protein–glycan interactions. To date, advanced and versatile NMR, X-ray crystallography, and MS methods [36–38] above all, have been developed to reach extensive information on the structural and conformational features of glycans and proteins. The experimental techniques employed for the analysis of these complex biomolecules are not discussed here; for a more in-depth understanding on this topic, the reader is referred to some comprehensive reviews [7,36,39–41]. Here, we focus instead on different computational and bioinformatic tools, designed to guide the structural and conformational elucidation process, and on the application of molecular dynamic simulations to the study of proteins and glycans in free and bound states. Detailed protocols and methods for protein and glycan modelling are extensively described and links to web servers and downloadable software, which can help researchers in designing the workflow to study a glycan–protein system, are also reported.

### Computational tools to study glycans in the free state

Since the first molecular dynamics simulations performed in the late 1980s on oligomannose type glycans [42] and in the early 1990s on complex type glycans [43], great steps forward have been made in the computational analysis of complex carbohydrates. The advancement of computing power, the emergence of GPUs, and specialised processors accelerated MD simulations making it a key scientific tool to explore complex systems, including glycans, with ever-increasing accuracy and efficiency [44].

#### Tools for building structural models of carbohydrates

Before going into details of the computational tools that can be used to dissect the 3D conformational features of glycans, an overview of the most useful web services and software to build 2D and 3D models of carbohydrate structures is reported here.

**Figure 3:**
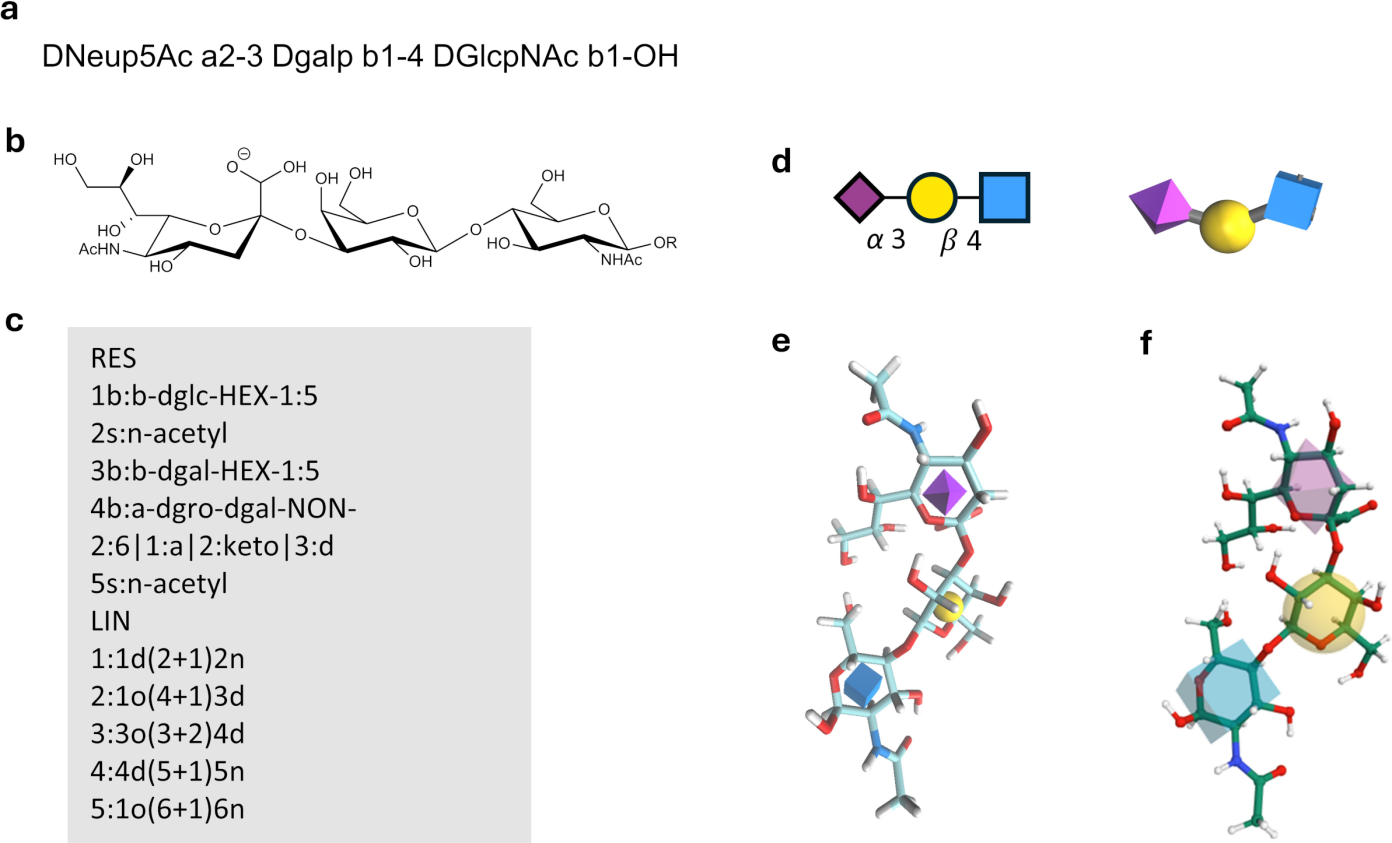
Different glycan representations. The 3’-sialyllactosamine is depicted according to the a) IUPAC nomenclature b) chemical representation as implemented in ChemDraw suite [46] c) GlycoCT nomenclature d) SNFG and 3D SNFG nomenclature e) VMD 3D-SNFG plugin f) MolStar representation.

Notably, despite the existence of several encoding formats for glycans (Figure 3), significant efforts have been made in the years to enable a simple and standardised glycan representation, which would simplify the transmission and efficiency of the communication within the scientific community. This led to the extensive use of the symbol nomenclature for glycans (SNFG) representation that is used in all the tools described below (Figure 3) [11,45].

Numerous computer applications have been developed to allow manual drawing and sketching of carbohydrates, as reviewed by Lal et al. [47]. Here, we list free tools useful not only for sketching and drawing but also for building the glycan structure of interest (last accessed date: May 2024) [47–48].

1. doGlycans [49]: Free desktop software package in the python framework that allows users to prepare carbohydrate structures for atomistic simulations of complex glycoproteins, glycolipids and carbohydrate polymers in the GROMACS force field format (see below). Polysaccharides can be prepared by using the *prepreader.py* tool, glycoproteins and glycolipids by using the *doglycans.py* tool. (https://bitbucket.org/biophys-uh/doglycans/).

2. Glycam-Web carbohydrate builder [50]: Free online web-service that gives the possibility to model the 3D structures of molecules and complexes containing carbohydrates starting from monosaccharide building blocks, being also able to add branching points and some sugar derivatisation, including methylation and acetylation. The user has also the possibility to choose the ring type and the anomeric configuration of each monosaccharide. Once the structure is complete, it is possible to download not only the generated .pdb files of the minimised resulting structures but also files for input to an AMBER simulation (https://glycam.org/). Notably, among the currently available interfaces for modelling oligosaccharide conformations on glycam website, one is dedicated to GAG modelling [51] (https://glycam.org/gag/).

3. CHARMM-GUI [52]: Online free web-service that offers a great variety of possibilities for reading and modelling .pdb files. It is a versatile program for atomic-level simulations, which can be run directly in the webserver. It has a special focus on macromolecules of biological interest; indeed, this platform contains a number of different modules designed to construct complex glycans, glycoconjugates as lipopolysaccharides (LPS), or even building a membrane system or solvating a protein. To use all these features, grouped in the *Input Generator* tab (https://www.charmm-gui.org/), it is necessary to be registered to the web site.

4. Azahar [53]: freely available python-based plugin that permits to visualise, analyse and model glycans and glycoconjugates (https://pymolwiki.org/index.php/Azahar).

5. 3D-SNFG: It is a script integrated in the visual molecular dynamics (VMD) program [54] (see below) that allows a cartoon representation of glycans according to the symbol nomenclature for glycans (see Figure 3) (https://glycam.org/docs/othertoolsservice/downloads/downloads-software/index.html).

#### Choosing the most appropriate simulation software package

With the aim to accurately prepare glycans for MD simulations, it is fundamental not only to build their 3D structure but also to choose the appropriate force field, that is a set of empirical energy functions and parameters used to calculate the potential energy of a system as a function of the molecular coordinates. The collection of equations and associated constants designed to reproduce the molecular geometry and selected properties of a system, as well as the naming and labelling of the system atoms, vary from one force field to another; therefore, it is important to ensure compatibility between the input file of the tested structure and the chosen force field. The Automated Topology Builder (ATB) and repository [55] (https://atb.uq.edu.au/) is a free web server providing topologies and parameters for a wide range of molecules. It provides access to classical force fields in formats compatible with different simulation packages, including GROMACS (see below), even offering a GROMOS to AMBER topology file converter. In the years, different MD simulation software packages have been developed and designed to simulate the movements and interactions of atoms and molecules over time; the three described below are currently widely used in the field of computational chemistry and biochemistry:

1. AMBER [56]: AMBER (https://ambermd.org/) is the acronym for "Assisted Model Building with Energy Refinement", and it is an open-source software widely employed for molecular modelling and simulation. It is known for its stability, user-friendly interface, and a wide range of analysis tools for studying complex biomolecular systems. AMBER provides various force fields, specifically optimised for simulating biological molecules, as lipids (lipids21) [57], proteins (ff14SB) [58], water molecules (TIP3P) [59], general organic molecules (gaff2) [60], and sugars (GLYCAM_06j) [61]. Notably, as mentioned above, on the GLYCAM-web site, it is possible to easily construct glycans with GLYCAM force field nomenclature; however, it is worth to note that only a few bacterial monosaccharides are available in the GLYCAM-web carbohydrate builder. For most bacterial sugars, the parametrisation of each building block is needed and requires the use of ab initio methodologies, including several steps of charges and electron density calculations, optimization and minimization, making the computational study of bacterial glycans difficult and time-consuming.

2. CHARMM [62]: CHARMM, acronym of "Chemistry at HARvard Molecular Mechanics", is a free extensively utilised molecular modelling and simulation software package. Its force field is at the core of CHARMM's capabilities, which serves as a comprehensive set of parameters and mathematical functions to describe the potential energy and interatomic interactions within a molecular system. The CHARMM force field includes parameters for various types of atoms, bonds, angles, dihedrals and non-bonded interactions, encompassing van der Waals forces and electrostatic interactions. CHARMM19 (united atom), CHARMM22, CHARMM27, and CHARMM36 (all atom) are some of the popular force fields available in the program. (https://www.academiccharmm.org/)

3. GROMACS [63]: GROMACS is the acronym for "Groningen Machine for Chemical Simulations"; it is a powerful open-source software package for molecular dynamics simulations in the field of computational chemistry and bioinformatics. It is extensively used to model and simulate the dynamic behaviour of various molecular systems, including proteins, nucleic acids, lipids, and small molecules. GROMACS provides various tools for system preparation, simulation setup, and post-simulation analysis. It is possible to use different force fields that include GROMOS96, GROMOS53A6, and GROMOS54A7, which are suitable for simulations of biomolecules, organic compounds, and a wide range of solvents (https://www.gromacs.org/index.html).

Despite several carbohydrate-specific force fields have been developed over the years [64–68], to date, the most widely used force field for carbohydrates is GLYCAM, which is continuously updated and improved to accurately describe their peculiar and complex set of conformational and energetic properties [61,69]. For specific studies involving unusual ligands, useful tools can be employed to provide the parameters needed for running MD simulations. Charge calculations and electron density computations for glycan units can be performed using tools like the online RED Server [70]. Although information on force fields is usually available, modifications can sometimes be required and can be achieved through ab initio calculations or programs like for example VFFDT [71].

#### Tools for the conformational analysis of glycans in the free state

The structure and biological functions of glycans are closely intertwined; the roles they play are influenced not only by their chemical composition but also by their conformation. As mentioned above, glycans are characterised by a huge conformational diversity (see Figure 1): even individual furanoid or pyranoid monosaccharides can assume various shapes and in longer glycans the relative orientation of the different monosaccharide building blocks is dictated by the values of different glycosidic torsion angles.

**MM calculations:** Investigating the energetically favourable conformations of carbohydrate disaccharide units composing the molecule of interest represents a pivotal step for generating reliable 3D glycan structure. A first analysis of glycan conformational features can be done by means of molecular mechanic calculations that allow to build the adiabatic energy maps, represented as a function of Φ and Ψ torsion angles, in which the energetic minima that can be populated by a specific disaccharide are reported [72–74]. Currently, different databases, which are described below, collect adiabatic energy maps facilitating the construction of glycan 3D models by enabling the selection only of permitted low energy conformations:

1. CSDB [75–77]: Carbohydrate Structure Database is a publicly accessible platform for multiple glycoinformatic studies and web tools, which among the other services allows the users to locate adiabatic maps for specific glycosidic linkages. Generally, CSDB offers a wealth of valuable features; it provides structural, bibliographic, taxonomic, NMR spectroscopic and other information on glycan and glycoconjugate structures of prokaryotic, plant and fungal origin. The retrospective literature analysis is the main source of structural data, which are then manually curated and approved. Besides structures, the database includes bibliography, abstracts, keywords, biological source data up to strains, methods used to elucidate structures, NMR signal assignment and other information (http://csdb.glycoscience.ru/database/).

2. Disac3DB: free annotated database that contains the 3D structural information of about 120 entries of disaccharides. For each disaccharide, an exhaustive search was performed using the MM3 molecular mechanics force field [66], giving a complete sampling of the conformational space and yielding the construction of relaxed adiabatic energy maps (https://glyco3d.cermav.cnrs.fr/disac3db/). It is worth to note that the presence of additional residues in the neighborhood of the studied glycosidic linkage may cause shifts in the values of the favored torsional angles. Thus, to evaluate if the presence of further residues results in limitations of the possible conformations of an individual glycosidic linkage, and/or if the adiabatic map of interest is not present in the aforementioned databases, the Schrodinger Suite of programs through the Maestro graphical interface can be exploited to generate the maps by using the MM3 force field [66]. The Schrodinger platform (https://www.schrodinger.com/) offers several services for molecular design and discovery providing access to physics-based molecular modelling tools and machine learning technologies from a single modelling environment, however, it is not free-accessible.

Further valuable insights into the structure and conformation of saccharides, determined by experiment and simulation, are available on the Stenutz's website (https://www.stenutz.eu). In particular, information on the preferred conformation of glycosidic linkages and the favoured dihedral angles for the OH group at position 6 in hexoses are reported. This website also provides a compilation of standardised procedures, providing practical guidelines for carbohydrate structural analysis, spanning from the purification to the structural analysis of polysaccharides.

**MD simulations:** Once the 3D glycan structure is built, taking into account the energetically favourable conformations of each constituent disaccharide unit, and the appropriate force field/simulation package is chosen, molecular dynamics simulations can be performed to gain insights into the glycan conformational behaviour. MD simulation generates an ensemble of conformations by applying the laws of motion to the atoms of the molecule [48], allowing to: i) sample the glycan conformational space; ii) investigate how the glycan behaves in a solution (if the MD is performed in explicit solvent), describing carbohydrate–water interactions; iii) monitoring the intramolecular interactions. Usually, this information has to be further validated by performing experimental studies (primarily nuclear Overhauser effect (NOE) and residual dipolar coupling-based experiments) to get accurate information on the glycan conformational behaviour and eventually apply some experimentally derived constraints.

The calculation and analysis of MD simulations of glycans in the free state can be performed with the same tools described below in the protein–ligand interactions section.

### Computational tools to study proteins in the free state

Knowledge on the three-dimensional structure of a protein is essential for understanding the functions and the dynamics of protein interactions. Several experimental techniques, including NMR, X-ray crystallography and Cryo-EM, can provide critical information for the characterisation of protein structure and conformation. Their widespread and wisely use permitted to experimentally determine the structures of around 200,000 proteins [78], all organized in the Protein Data Bank (PDB), that is a freely and publicly available central archive of macromolecular structural data, established in 1971. However, the three-dimensional shape of billions of known protein sequences is not available yet. In this scenario, bioinformatic tools can come to the aid of predicting protein three-dimensional structure with high accuracy, as outlined below [79–85]. Notably, generated models have also occasionally helped solve protein structures [86], further highlighting the great potential of the integrated use of bioinformatic tools and experimental data.

#### Tools for protein structure prediction

Due to the vast conformational space and a complex energy function, protein structure prediction (PSP) is a computationally challenging task. Homology modelling is a template-based PSP that may be used to predict the 3D structure of a protein based on its amino acid sequence and the structure of a related protein that is already known. However, also template-free PSP has obtained significant progress recently via machine learning and search-based optimisation approaches [87]. There are several software programs and tools available for homology modelling, and some of the most popular include:

1. AlphaFold2 [88]: It is an open-access protein structure prediction system based on artificial intelligence and machine learning. It is based on a neural network that can predict the 3D protein structure at a high accuracy level. The AlphaFold solution is composed of two steps. First, given a protein sequence, it generates multiple alignments with sequences from all the species, including evolutionary profiles from different sources. In the second step, a model refinement is generated based on structural refinement (where the network optimises the torsion angles, bond length and bond angles), distance constraints (according to laws of physics) and gives an output with the structure with the minimised energy (https://alphafold.ebi.ac.uk/).

2. I-TASSER [89]: Iterative Threading ASSEmbly Refinement is a free online server that combines ab initio protein structure prediction with template-based modelling. It is known for its ability to predict both the structure and function of a protein. It is based on identifying structural templates from the PDB by several threading methodologies with full-length atomic models (https://zhanggroup.org/I-TASSER/).

3. Modeller [90]: It is an open-access program used for homology or comparative modelling of proteins. The user inputs an alignment of a sequence to be modelled with known related structures, and the computer generates a model of all non-hydrogen atoms. It can do de-novo modelling of protein loops and apply spatial constraints (https://salilab.org/modeller/).

4. Rosetta [91]: It was developed for de novo protein structure prediction in a free version. Homology modelling is also applied in this instance by using several protein templates that hybridise the most homologous sections of various templates into a single model while modelling missing residues de novo. Advances in the scoring function, which is a mix of physics-based and knowledge-based potentials fitted against known structures and thermodynamic observables, have increased the accuracy of predictions. Incorporating experimental data into models has been made more accessible. The same research group also developed RoseTTAFold, which uses deep learning to quickly and accurately predict protein structures based on limited information [92]. However, very accurate structures for complex proteins are yet to be achieved at a level suitable for effective drug design. Moreover, ab initio prediction of a protein's structure only from its amino acid sequence remains unsolved. Accessing Rosetta molecular modelling software tools (https://www.rosettacommons.org/software) has traditionally required expertise in the Unix command line environment, limiting their use. A web server called ROSIE [93] was created to provide a more accessible environment for selected Rosetta protocols. Academic users can access ROSIE freely (https://rosie.rosettacommons.org/).

5. SWISS-MODEL [94]: It is a web-based integrated free service dedicated to protein structure homology modelling. It guides the user in building protein homology models at different levels of complexity. This program builds a homology model by employing four main steps: (i) identification of structural template(s), (ii) alignment of target sequence and template structure(s), (iii) model-building, (iv) model quality evaluation. Each of the above processes may be repeated interactively until a satisfactory model is produced (https://swissmodel.expasy.org/).

6. UniLectin [95]: It is an interactive, publicly accessible platform that provides curated and predicted lectin data, not only including structural information on lectins and their interactions with carbohydrate ligands, but also predicting the occurrence of lectins in genomes. UniLectin3d is one of the modules integrated in UniLectin, which provides curated information on 3D structures of lectins [94–96] a classification system based on both taxonomic origin and structural fold (https://unilectin.unige.ch/).

7. GlycoShape3D [97]: It is a freely available database for academic user that enriches the landscape of glycobiology resources. It offers structural insights into glycoproteins, addressing challenges posed by glycan complexity, flexibility, and heterogeneity. In particular, the Re-glyco tool allows the user to restore the missing glycosylation on glycoproteins deposited in the RCSB PDB or in the EBI-EMBL AlphaFold protein structure database (https://glycoshape.org/).

The quality of the generated protein model is contingent on elements such as the chosen template structure (if any), the sequence alignment, and the choice of the modelling algorithm. To ensure a high accuracy of the predicted model, which should be at least comparable to that of experimental structures, several programs can be employed for model validation and refinement. Among them, PROCHEK [98] is an open-source program that permits to check the quality of the protein structure by analysing the Ramachandran plots, the planarity of peptide bonds, the bad non-bonded interactions, the distortions of the geometry around the Cα atoms, the energies of hydrogen bonds, and the departure of the side chain χ torsion angles from expected values. Improvement and/or validation of modelled or experimentally solved structures can be also obtained by using CASP (critical assessment of protein structure prediction) [99], which consists of a free platform established in 1994 to help advance the methods of identifying protein structure from sequence. The Protein Structure Prediction Center (https://www.predictioncenter.org/) has been organized to allow researchers to objectively test their structure prediction methods. Some of the best performing methods (including among the others AlphaFold, RosettaFold and I-TASSER) are implemented as fully automated servers, which can be used by public for protein structure modelling.

**MD simulations:** Generating an accurate protein model or choosing the appropriate published 3D structure of a protein is essential to obtain reliable and precise results from MD simulation. It is also worth to note that, to generate the input files for MD, some modifications on the .pdb file of the protein are required. For instance, capping the protein termini with non-charged groups and replacing original hydrogens to guarantee compatibility with the selected force field is required before running MD simulations. Other modifications can include adding a disulfide bond between specific cysteines and filling the missing side chains and missing loops (if any) to restore the integrity of the protein. Here, we list a series of software tools and packages which are commonly employed to generate the protein input files for MD:

1. Molprobity [100]: It is a widely used web-based software suite for evaluating and enhancing the quality of protein structures, especially those intended for molecular dynamics simulations, available in a free version. Specifically, it is possible to check the H atoms, the quality of the structure, evaluate some steric clashes and visualise in a friendly manner the full structure (http://molprobity.biochem.duke.edu/).

2. PDBtools [101]: It is a freely accessible software that allows the manipulation and modification of a PDB file. Different tools are available, such as deleting atoms, renaming the polypeptide chain, calculating disulphide bonds, adding missing atoms and mutating residues (https://wenmr.science.uu.nl/pdbtools/submit).

3. ProteinPrepare [102]: This application enables users to modify PDB files and create input files for molecular dynamics by adding missing atoms, removing H atoms, and analysing the proton state of amino acids. The registration of the user to the web-site is required to access these tools (https://playmolecule.com/proteinPrepare/).

Several MD simulation packages, including CHARMM-GUI [52], AMBER [56], and GROMACS [63], offer built-in utilities for preparing input files. These tools also provide extensive documentation and tutorials to help users effectively create MD input files for proteins. Once the protein input files are generated, MD simulations can be run.

### Computational tools to study protein–ligand complexes

Detailed investigations of protein–ligand interactions, combining experimental and computational methods, provide an indispensable basis to depict holistic pictures of molecular complexes allowing to modulate them at will. The computational approach involves i) predicting/building the protein and the ligand in their optimal conformation (as discussed above), ii) predicting the protein binding site; iii) modelling the ligand into the protein binding site, iv) assessing binding affinity through sampling and scoring, as discussed in the following paragraphs [103].

#### Prediction of the protein binding site

Over the years, structure-, sequence-, and homology/template- based methods have been employed to identify and predict carbohydrate-binding sites starting from the protein structure [104]. Recently, thanks to the fast development of machine learning techniques, new computational tools have been developed to facilitate the prediction of protein binding sites.

We report here only the applications related to the protein interaction with glycans:

1. PeSTo-Carbs [105]: it is an extension of Protein Structure Transformer (PeSTo) [106], a deep learning method to predict protein interaction interfaces with other proteins, nucleic acids, lipids, small molecules, and ions, starting from a protein structure. PeSTo-Carbs is specifically trained to predict carbohydrate and cyclodextrin binding interfaces on proteins. Two different modules are available: a general model PS-G for a wide range of carbohydrates, their derivatives and cyclodextrins, and a specific model PS-S for important carbohydrate monomers. All of these features are available for free without registration as online tools (https://pesto.epfl.ch/).

2. GlyNet [107]: it is a free deep learning algorithm, based on neural networks (NN), that allows the user to predict protein-glycan binding. Taking a glycan structure as input, this model is able to predict the strength of the interaction based on the relative fluorescence units (RFUs) measured in the Consortium for Functional Glycomics glycan arrays and extrapolating these to RFUs from untested glycans (https://github.com/shauseth/glynet).

3. LectinOracle [108]: it is a freely available deep learning-based model that combines transformer-based representations for proteins and graph convolutional neural networks for glycans to predict their interaction (https://github.com/BojarLab/LectinOracle).

4. CAPSIF [109]: CArbohydrate-Protein Site IdentiFier is a convolutional neural network able to predict protein–carbohydrate binding interface from a protein structure. In contrast to other DN algorithms, as GlyNet and LectinOracle, which predict lectin-carbohydrate binding on a protein level, it provides residue-level information for non-covalently bound carbohydrates either from an experimental or generated-model protein structure. It includes two modules: CAPSIF-Voxel that predicts the protein binding residues and CAPCIF-Graph that predicts which residues bind sugars. It is freely available for use, and the code for CAPSIF can be accessed on GitHub (https://github.com/Graylab/CAPSIF).

To identify potential binding sites on the protein's surface, docking calculations can also be performed (see below).

#### Docking calculation tools for interaction studies

Molecular docking plays a crucial role in computer-aided drug development, allowing systematic evaluation of compound libraries to identify high-affinity lead compounds for specific targets. Bio-algorithms enable modelling protein tertiary structures, predicting ligand binding pockets, and supporting drug discovery through molecular docking [110]. Advances in information technology and improved computational efficiency have made computational methods integral to modern biological research, and large-scale structure-based docking screens have become common, facilitating the exploration of vast chemical spaces and identifying potential target hits from extensive compound libraries [103]. While docking programs and servers may exhibit variations in their operational methods, they generally adhere to a common workflow comprising two primary phases. The first phase involves a conformational search aimed at predicting potential ligand conformations. This is followed by the second phase, which focuses on scoring the binding poses obtained during the conformational search. In this phase, the generated ligand–receptor complexes are assessed and ranked based on their binding energy thanks to the use of scoring functions [111].

Docking calculations can be conducted in two distinct ways: blind dockings, which explore the entire protein surface [112], and directed docking, typically employed when prior knowledge of the binding pocket exists and performed within a predefined box. Blind dockings are performed using cavity detection programs and online servers, as follows:

1. CB-Dock2 [113]: Cavity-detection guided Blind Docking 2 (https://cadd.labshare.cn/cb-dock2/index.php) is an online protein–ligand docking program designed to perform blind docking at predicted sites instead of the entire surface of a protein. Thus CB-Dock automatically recognises putative binding sites to determine their centre and size, with the aim to adjust the docking box to suit specific query ligands. Finally, molecular docking calculations are performed with Autodock Vina (see below).

2. Fpocket [114]: It is an open-source pocket detection package based on Voronoi tessellation and alpha spheres. It consists of three main programs: Fpocket for pocket identification, Tpocket for benchmarking pocket detection, and Dpocket for collecting pocket descriptor values. Written in C, Fpocket is well-suited for developing new scoring functions and extracting various pocket descriptors on a large scale. Fpocket 1.0 outperforms industry standards by detecting a high percentage of pockets within the best-ranked ones and offers a fast, open-source solution for protein pocket detection (https://github.com/Discngine/fpocket).

3. GRID [115]: It is a computational tool used to identify energetically favourable binding sites, known as molecular interaction fields (MIFs), on molecules with known structures. GRID has various applications, including ADME prediction, site of metabolism prediction, ligand-based and structure-based design, pharmacophore elucidation, water network prediction, and 3D-QSAR. GRID 2021 introduced a new interface aimed at structure-based design. It enables users to explore binding sites using classic GRID MIFs, encompassing 74 different chemical types. Additionally, it offers a new molecular probe for generating MIFs specific to fragments of interest. GRID 2021 includes a 3D sketcher for visualising ligand modifications, and its Designer mode assists in finding optimal chemical moieties for specific sites (https://www.moldiscovery.com/software/grid/#:~:text=GRID%202021%20is%20a%20new,MIFs%20for%20fragments%20of%20interest).

When there is prior knowledge of the protein binding pocket, it is time saving to define an optimal docking search space or box and study specific binding pockets, improving docking accuracy and efficiency. Customising the box size for individual ligands, based on their size and the relationship with the search space, can be done by comparing the target protein to related proteins or those co-crystallised with ligands [103] and manually superimposing the new ligand to the reference structure. Some payment software like Glide [116–117], GOLD [118] and Molecular Operating Environment (MOE) dock [119] can be used for this purpose, although here are listed free docking tools:

1. Autodock [120–121]: It is a suite of advanced docking tools used for predicting how small molecules, such as drug candidates or substrates, interact with known 3D protein structures. It offers two generations of software, namely AutoDock 4 and AutoDock Vina, and a user-friendly graphical interface called AutoDockTools (ADT) to assist in configuring ligand rotatable bonds and analysing docking results. Additionally, the accelerated AutoDock-GPU is designed for faster performance, surpassing the original single-CPU docking code by hundreds of times. AutoDock 4 comprises two main programs: *autodock* handles ligand docking by aligning it with precomputed protein grids, while *autogrid* generates these grids. The grids can also assist organic chemists in designing better binding molecules (https://autodock.scripps.edu/).

2. Autodock Vina [122]: It is the open-source improved successor of Autodock. Vina is improved in terms of accuracy and performance as simplifies the process by instantly calculating grids internally, eliminating the need for manual grid map selection and atom type assignments (https://vina.scripps.edu/#).

3. FlexAID [123]: It is a molecular docking software capable of setting small molecules and peptides as ligands, and proteins and nucleic acids serve as docking targets. Notably, FlexAID shows support for full ligand flexibility and the flexibility of side chains in the target. It achieves this by employing a soft scoring function that assesses the complementarity between the surfaces of the ligand and the target. Thus, FlexAID has demonstrated superior performance compared to well-established software like AutoDock Vina, particularly when target flexibility plays a pivotal role, as is often the case when working with homology models (http://biophys.umontreal.ca/nrg/resources.html).

4. HADDOCK [124]: High Ambiguity Driven protein–protein DOCKing is an advanced computational approach used for modelling interactions in biomolecular complexes. Noteworthy, HADDOCK incorporates information from known or predicted protein interfaces into the docking process through ambiguous interaction restraints and allows the specification of precise distance restraints (e.g., based on MS cross-links). It also supports a range of experimental data, including NMR residual dipolar couplings, pseudo contact shifts, and cryo-EM maps, positioning HADDOCK as a versatile tool capable of handling various modelling scenarios, such as protein–protein, protein–nucleic acids, and protein–ligand interactions.

The majority of existing docking software was originally designed for small, rigid, drug-like molecules, therefore, limiting their effectiveness in studying protein–carbohydrate interactions [125–126]. The development of specialized programs has been crucial in enhancing the accuracy of docking calculations [125–126]. We here listed a series of programs for running docking calculations with a special focus on those specifically designed to address the unique challenges posed by glycans [127–128].

1. Vina-Carb [129–130]: Vina-Carb is a module of AutoDock Vina (downloadable with a free version at https://glycam.org/docs/othertoolsservice/downloads/downloads-software/index.html), proven to be a valuable tool for studying carbohydrates. It incorporates carbohydrate intrinsic (CHI) energy functions and explicit water to better handle glycosidic linkages and improve docking accuracy. When Vina-Carb was applied to antibodies, lectins, and carbohydrate binding modules (CBM), the success rates in predicting accurate binding modes reached 86%, 50%, and 42%, respectively, compared to 70%, 50%, and 0% for AutoDock Vina. Although Vina-Carb generally performed slightly better over AutoDock Vina when docking glycans to proteins, it does not always rank the best docking pose as the top scoring pose.

2. BALLDock/SLICK [131]: It is a molecular docking method specifically designed to accommodate carbohydrate-like compounds, employing a genetic algorithm that allows for ligand and receptor side-chain flexibility. Designed specifically for protein–carbohydrate interactions, SLICK includes terms that consider CH–π interactions, hydrogen bonds, smoothed van der Waals interactions, and electrostatic interactions. The SLICK scoring function, tailored for carbohydrates, enhances the accuracy of predicting binding modes and free binding energies. Compared to other programs such as AutoDock and FlexX (see below), BALLDock/SLICK demonstrates superior performance in structural and energetic precision. This method is particularly valuable in drug design involving protein–carbohydrate interactions, addressing weaknesses such as the CH–π interactions that are challenging for other programs like Vina-Carb.

3. FlexX [132–133]: It is a molecular docking software (unfortunately not free) designed to predict the conformations of small molecules in protein binding sites, thus facilitating the discovery of new drugs. Within SeeSAR, its functionality allows ligands to be placed in binding sites using an incremental construction algorithm that splits ligands into fragments, places, and scores them quickly in the binding site. The best fragments are then assembled to form the complete ligand, optimizing the generated conformations. FlexX's strengths include rapid and efficient exploration of the conformational space, handling ligand flexibility, a precise scoring function, and smooth integration with other molecular modelling programs. Additionally, FlexX excels in processing large libraries at high speed and is user-friendly, requiring no prior receptor preparation.

4. ROSETTA [134]: The development of GlycanDock [134], a protein−glycoligand docking refinement algorithm integrated in the RosettaCarbohydrate framework [135], allowed the use of Rosetta macromolecular modelling and design software suite to perform docking calculations on glycans bound to proteins with a higher accuracy with respect to previous Rosetta’s protein−small molecule docking algorithms. Unlike other docking programs such as AutoDock, AutoDock Vina, DOCK, FlexX, Glide, and GOLD, which are primarily designed for small, rigid ligands, GlycanDock is specifically optimized to address the flexibility and complex structural features of glycans. The carbohydrate chains are treated as flexible oligomers, allowing extensive conformational sampling of the glycoligand while maintaining glycosidic linkages within predetermined, energetically favorable minima to ensure biophysically realistic carbohydrate structures. GlycanDock handles the flexibility and complexity of glycans better than other docking programs and can be downloaded as part of the Rosetta package from the Rosetta Commons (https://www.rosettacommons.org).

5. GlycoTorch Vina [136]: GTV is a free molecular docking tool specifically designed for GAGs. Based on Vina-Carb, it enhances the accuracy of modeling these carbohydrates by including parameters for sugars in the 2SO conformation and glycosidic linkages specific to GAGs. GlycoTorch Vina also allows the integration of experimental data, such as NMR, and considers water-mediated interactions, providing more accurate predictions in the formation of GAG-protein complexes.

6. DOCK [137]: It is a molecular docking program (free for academic research) that predicts the orientation and conformation of ligands within the binding site of proteins or nucleic acids. It uses an incremental construction approach ("anchor-and-grow") to handle ligand flexibility and employs a scoring function based on the AMBER force field to evaluate the stability of the complex. DOCK is particularly useful for GAGs due to its ability to accurately model conformational flexibility and enhance sampling, allowing for more precise predictions of ligand–receptor interactions.

7. ATTRACT [138]: It is a docking (not free) program that models interactions between proteins and other biomolecules such as DNA, RNA, and small ligands. Originally designed for protein docking, it has been successfully adapted for GAGs due to its coarse-grained force field approach, which allows for protein flexibility and the simultaneous handling of multiple protein bodies [139]. This adaptability makes it particularly useful for large and dynamic complexes. Although not initially intended for GAGs, researchers have modified its protocols to account for the unique features of these molecules, such as their high flexibility and electrostatic charge. This has enabled ATTRACT to effectively predict binding poses and rank GAG-protein complexes, demonstrating its utility and versatility in advanced biological interaction studies.

The key distinctions among various docking programs stem from the specific computational search algorithms they employ and the characteristics of the scoring functions utilised to order the docked poses. Over the years a plethora of different scoring functions have been developed, in particular thanks to the evolution of machine learning and collection of high-resolution structural information [140–142]. Recently, some of them have been also optimised for evaluating the binding affinities between proteins and carbohydrates [143–144]. Among them, the CSM (cutoff scanning matrix)-carbohydrate outperforms previous methods and scoring functions, also providing a freely accessible and user-friendly web interface and an application programming interface (API) (http://biosig.unimelb.edu.au/csm_carbohydrate/).

#### Unravelling complex molecular interactions: tools for molecular dynamics studies

Once the conformational space accessible to the ligand has been studied, the protein binding pocket has been identified, and a model of protein–ligand complex has been obtained, MD simulations can be performed with the aim to accurately describe the conformational and dynamic properties of the bound state. All-atom MD simulations involve 4 steps: i) energy minimisation, ii) heating, iii) equilibration and iv) production. MD simulations are often run by using explicit solvent box to account for molecular interactions, and if needed, Cl^−^ or Na^+^ ions are added to neutralise the system. Although different programs for running MD simulations, including CP2K [145], DESMOND [146], LAMMPS [147], TINKER [148], YASARA [149], and NAMD [150] are available, the most widely used packages to run all-atom MD simulations on protein glycan complexes are the already described AMBER [56], CHARM-GUI [52] and GROMACS [63].

#### Tools for the analysis of computational data

Once the protein in the apo-form has been analysed, the ligand in the free-state has been studied, and the protein–ligand complex has been extensively subjected to MD simulations, in-depth insights into the structural and conformational features governing the molecular interactions can be achieved by analysing and visualising the obtained data. The critical step of post-processing analysis involves examining the binding poses of the ligands, evaluating the stability and dynamics of the complexes, investigating the electronic structure and interactions and calculating binding energies or free energy profiles within the system, thus helping in understanding the energetics and thermodynamics of the interactions [151]. To facilitate these tasks, various software tools have been developed; generally, the simulation packages used to run all-atom MD simulations offer built-in utilities and scripts for post-processing the MD data. For example, AmberTools, released within the AMBER suite of biomolecular simulation programs, includes cpptraj and ptraj codes for analysing structure and dynamics in trajectories [152]. Additionally, other programs as VMD (see below) and PLUMED [153], an open-source library compatible with popular MD engines like Amber and GROMACS. (https://www.plumed.org/), can support data analysis for molecular dynamics simulations.

Moreover, several custom scripts have been developed within computational chemistry laboratories (see for example: https://github.com/roviralab/utils) enabling the tracking of glycan conformational changes throughout the dynamics, monitoring dihedral angles, distances, and other parameters. Additional programs allow for the combination and comparison of experimental and theoretical data, enhancing the reliability and accuracy of the simulations. As example, the software package MD2NOE [154] permits to properly simulate NOE effects also of flexible molecules sampling multiple conformational states directly from molecular dynamics (MD) trajectories. With the advent of GPU-based simulation code, indeed, MD simulations have been extended into the microsecond regime, allowing to sample glycan conformational space sufficiently and enabling the computation of key NMR properties [154].

**Figure 4:**
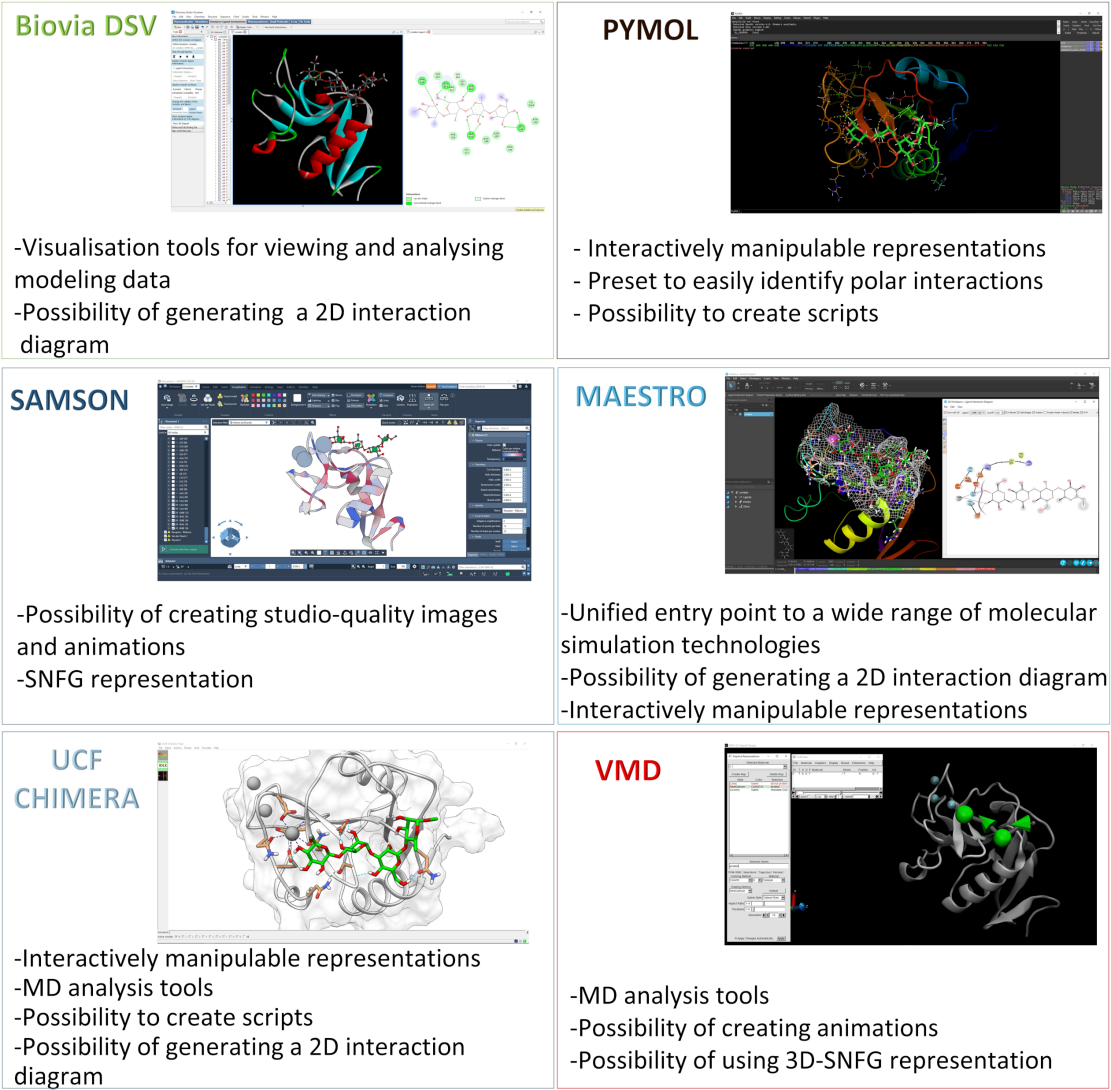
Visualisation programs. Different representation of a protein–ligand complex by using the most used visualisation programs reported in this review. The previously published complex [155] between the Ca^2+^ dependant C-type lectin, DC-SIGN (PDB: 1SL4) [156], and a tetrasaccharide composed of mannose and rhamnose residues, has been used to highlight the main advantages of each visualisation program applied to protein–glycan complexes.

Different visualisation programs, as those described below, play a crucial role in rendering complex 3D structures, visualising molecular interactions, and generating high-quality images for publications or presentations (Figure 4). These user-friendly tools are indispensable for researchers in the fields of structural biology, biochemistry, and computational chemistry, making it easier to comprehend and communicate the results of sophisticated simulations.

1. VMD [54]: Visual Molecular Dynamics is a popular and freely accessible molecular modelling program designed to display, animate, and analyse biomolecular systems using 3D graphics and built-in scripting. It provides tools for simulation preparation, visualisation, and analysis of molecular dynamics. (https://www.ks.uiuc.edu/Research/vmd/).

2. PyMOL [157]: It is an open-source molecular visualisation system developed by Schrödinger. It is one of the most used programs for the visualisation of the 3D structure of the protein alone or in a complex with a ligand. Several tools are available to create, manipulate and visualise the 3D structures. Other tools consent to generate the surface of a protein and highlight the electrostatic potentials or hydrophobicity. PyMOL is used for various applications, such as protein structure analysis, molecular docking studies, and drug design.

3. UCSF Chimera [158]: It is a highly versatile and widely used free molecular visualisation and analysis program developed by the University of California, San Francisco (UCSF). It is a powerful software tool for visualising and analysing the 3D structures of biological macromolecules, such as proteins, nucleic acids, and other complex molecular assemblies. UCSF Chimera provides a user-friendly interface for exploring and manipulating molecular structures, offering a wide range of features for tasks like molecular modelling, molecular dynamics analysis, structural biology, and more. UCSF Chimera is commonly used to gain insights into the structure and function of biomolecules. It supports various file formats, offers diverse visualisation options, and allows for the creation of stunning images and animations of molecular structures, making it an invaluable resource (https://www.cgl.ucsf.edu/chimera/).

4. BIOVIA Discovery Studio [159]: It is a freely downloadable suite of science applications designed for life sciences discovery research, which includes addressing multiple optimisation objectives in drug discovery. This comprehensive software suite, built on BIOVIA Pipeline Pilot, provides a wide range of validated applications. It offers a scalable and collaborative research environment, making it a valuable tool for life sciences discovery research (https://discover.3ds.com/discovery-studio-visualizer-download).

5. Schrödinger Maestro [160]: It is a comprehensive molecular modelling and computational chemistry software suite designed for researcher fields like drug discovery, materials science, and structural biology. Schrödinger Maestro provides tools for molecular visualisation, ligand-receptor docking (with Glide [117]), molecular dynamics simulations, quantum mechanics calculations, and more. It is widely used in the pharmaceutical and biotechnology industries for drug design and discovery, as well as in academic research and other scientific applications that involve the study of molecular structures and interactions (https://www.schrodinger.com/products/maestro).

6. SAMSON [161]: Software for Adaptive Modelling and Simulation Of Nanosystems is a computer software platform for molecular design, unfortunately not freely avaiable. Its modular architecture enables a wide range of tasks, including model creation, calculations, interactive or offline simulations, and result visualisation and interpretation. Notably, SAMSON offers modules related to glycans and glycans visual models, facilitating the use of the SNFG nomenclature for ligand design and visualisation (https://www.samson-connect.net/).

**Figure 5:**
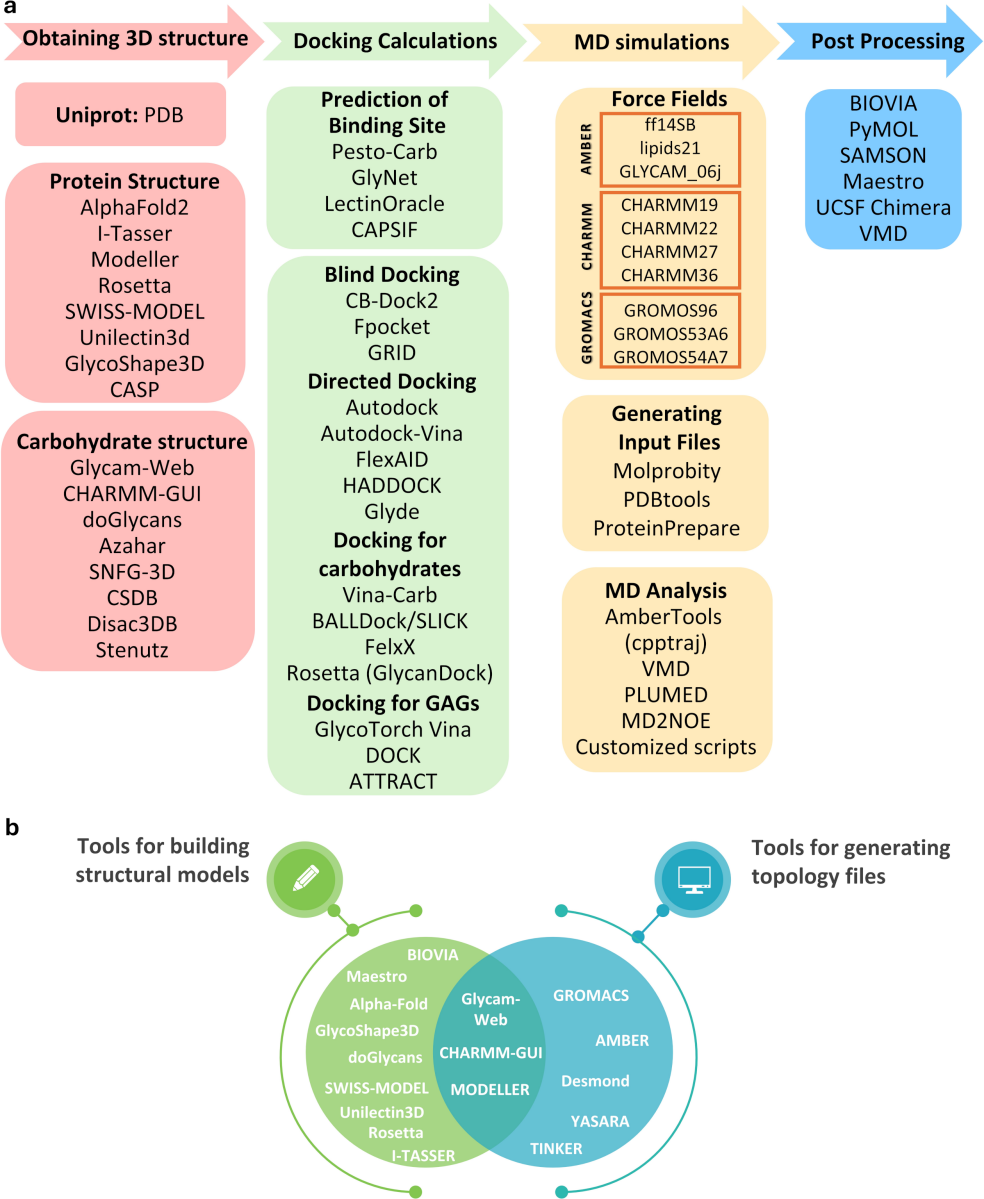
A schematic representation of useful computational methods to study protein–glycan interactions. a) Workflow including different key steps needed to analyse protein–glycan interactions: 1. Building/choosing the appropriate glycan/protein 3D structure; 2. Modelling protein–glycan complex; 3. Running and analysing MD simulations; 4. Processing and visualising the results. b) Summary of the presented tools for building structural model and/or generating topology files of glycan/protein structures.

All the computational tools here reported, summarised in Figure 5, constitute a unique kit for the analysis of protein–glycan interactions.

### Computational tools applied to the study of glycans in the free state

In the years, the architectural and conformational features of different mammalian glycans, including oligomannose [162] and complex-type *N*-glycans, have been unravelled by employing computational approaches. As example, the work conducted by A. M. Harbison et al. [163] reported the molecular dynamics of complex biantennary IgG Fc *N*-glycans and their implications for the structural integrity and functionality of human immunoglobulins G (IgGs).

MD methods have also been employed, in combination with experimental methods, as NMR, to explore the three-dimensional features of bacterial glycoconjugates, as the exopolysaccharides [164], and the rough-type lipopolysaccharide [165] isolated from *Methylobacterium extorquens* or the lipopolysaccharide isolated from *Herbaspirillum* Root189 [164]. In these studies, once built the parameters for non-standard bacterial monosaccharides, which were not included in the GLYCAM-website, the overall conformation and properties of the saccharide chain has been accurately described and compared to the experimental NOE data. The tight integration between computational and experimental results allowed to highlight how modifications of the saccharidic backbone, as example with *O*-methyl and *O*-acetyl groups, can affect the polysaccharide biophysical properties tuning its ability to interact with other polymers and/or receptor proteins.

Another example of the application of simulation methods to the analysis of complex glycans is presented by Makshakova et al., who analysed the three-dimensional structure of the exopolysaccharide isolated from *Alteromonas infernus* GY785 [166]. The main chain of the so-called “Infernan” polysaccharide includes glucose, galacturonic acid and galactose, with branches composed of uronic acids and sulphate groups that contribute to modulate its unique properties. Specifically, the authors used molecular mechanics and dynamics calculations to describe the helical structure of the polysaccharide chain and the role of its side chains in the creation of Ca^2+^ chelating sites in the region of the polysaccharide branching points.

A further application of computational tools to the analysis of complex glycans is the study of the conformational behaviour of the naturally cationic polysaccharide, carboxymethyl chitosan (CMCS), reported by Zhang et al. [167]. Due to its non-toxic, biodegradable, biocompatible, and versatile features, chitosan has been widely used in various fields such as biomedicine, cosmetics, agriculture and food. However, its insolubility in neutral or alkaline pH conditions largely limits chitosan's applications. MD simulations were thus employed to mimic the behaviour of CMCS in water under different pH values and different degrees of deacetylation and substitutions in order to study its aggregation pattern.

### Computational tools applied to the study of proteins in the free state

Understanding the dynamics of proteins in their free state is key to investigate how they can interact with other biomolecules. MD simulations can be used to study the conformational changes that occur in proteins as they move from one state to another, which is important for understanding their function; additionally, MD simulations can also be used to study the thermodynamics of protein folding, which is important for understanding how proteins fold into their native state.

Recently, extensive MD studies have been performed to investigate the structural and functional features of the SARS-CoV-2 spike glycoprotein, allowing to reveal the critical role of glycans attached to the viral protein in the infection. In particular, the full spike receptor consists of a trimer of S protein, and each monomer comprises 22 *N*-glycan sites. Recent studies suggest that the structure and occupancy of the SARS-CoV-2 S glycans affect the structural integrity of the trimer [168]. Specifically, different Spike protein models with varying glycan compositions in positions N234, N165 and N343 were created by homology modelling using SWISS-MODEL. Then *N*-glycans were added by aligning conformationally equilibrated *N*-glycan structures from a Glyco Shape library to the GlcNAc residues resolved in the cryo-EM structure, with adjustments of the torsion angles to resolve steric clashes with the surrounding protein. By using AMBER, the MD simulation was performed and suggested that diminishing the size of *N*-glycans at position N234 results in destabilising the "wide-open" conformation of the receptor-binding domain (RBD). This destabilisation leads to increased RBD dynamics. Furthermore, the composition of *N*-glycans at positions N165 and N343 influenced the stability of the open RBD, where shorter structures exhibited reduced effectiveness in interacting with the disordered loop within the receptor-binding motif.

Moreover, several groups have employed MD simulation to design new proteins. For example, the group of Mayo [169] employed this approach to engineer a de novo homodimer from a monomeric protein. The integration of computational protein design (CPD) and MD simulation allowed to refine the structural and dynamic aspects of the designed proteins overcoming. CPD inherent limitations, including constraints on side chain rotamers, fixed protein backbones, and a lack of consideration for solvent interactions. Thus, the use of MD simulation allowed to provide a more precise depiction of the protein's behaviour, shedding light on its dynamics and stability.

Another example of the relevance of all-atom molecular dynamics simulations is the study performed by X. Cao et al. [170], which reports the structural dynamics of GH33 sialidases. The computational analysis revealed significant conformational rearrangements within the enzyme active sites leading to the formation of a new cleft to accommodate glycosyl acceptors. Furthermore, the simulations shed light on the role of specific residues within the enzyme's active site, such as the arginine triad and other key residues, which adjusted their conformations to interact with sialic acid and facilitate the opening of a new cleft. Computational tools, including GROMACS and AutoDock, were pivotal in uncovering key insights into the catalytic mechanisms of GH33 sialidases offering a promising avenue for the rational design of improved biocatalysts.

### Computational tools applied to the study of protein–glycan interactions

Molecular dynamics simulation has proven to be a powerful tool in understanding and elucidating the intricate dynamics of glycan interactions with biomolecules. This computational technique allows researchers to delve deep into the molecular-level details of how glycans bind to their respective target proteins, providing valuable insights into binding mechanisms, thermodynamics, and the overall stability of protein–glycan complexes.

MD methods have been extensively employed by different groups to explore glycan recognition by host receptors, including mammalian and bacterial proteins. For example, several studies have been published on the recognition of sialic acids by different classes of proteins. It is known that sialic acid plays an essential role in the modulation of immune response through the binding with sialic acid-binding immunoglobulin-like lectins (SIGLEC). In the study reported by Martin Frank et al. [125], MD were used to analyse the binding modes of several glycomimetics for Siglec-7 and describe their molecular interactions at atomic level. The conformational characteristics of both natural, unmodified, and synthetic, modified α-sialoside glycerol sidechains of sialic acid were investigated. The applied computational tools allowed to discover a new modification in the sialic acid glycerol chain that binds to Siglec-7, providing a basis for designing next-generation Siglec-7 ligands.

Sialic acid can also be recognised from some bacterial proteins which exploit this interaction to adhere to host cells during the first stages of infection. An example is given by the sialic acid-binding serine-rich repeat adhesins from *Streptococci,* which contain a sialic acid-binding region (SLBR) and are known as Siglec-like adhesins. Di Carluccio et al. [171] described the interactions between two different siglec-like adhesins with natural glycans by using a combination of NMR and MD simulations. This integrated approach allowed to accurately describe the different selectivity and flexibility of the proteins towards sialoglycans recognition and binding, providing a privileged starting point for the design and development of novel compounds to counteract streptococcal infections by inhibiting bacterial adherence to host tissues.

Another example of the application of MD simulations in studying bacterial proteins in the interaction with glycans comes from Bernardi's group [172], whose focus was on examining the interaction between different glycomimetic antagonists and BC2L-C lectin derived from *B. cenocepacia*. The MD results showed that the binding site at the interface of two BC2L-C-Nt monomers is pre-organised to host the bifunctional ligands. Additionally, the simulation with the water molecules highlights the importance of two of these molecules in the binding site, establishing an interaction network.

Bacterial glycoconjugates, as lipopolysaccharides-related systems, have also been dissected, gaining critical information about the ability of LPS to both stimulate the host immune system, mainly by interacting with TLR-4/MD-2 complex, and interact with several molecules. The Martin-Santamaria group [173] extensively contributed to increase the knowledge on this topic, analysing the conformational changes of the TLR4/MD2 complex when interacting either with small and LPS-like molecules

Notably, the comparison of free and bound state MD results can allow to determine critical differences in the glycan conformational behaviour upon binding with selected proteins, paving the way for the design of tailored synthetic inhibitors and therapeutics. As example, in the study of L. Pirone et al. [174], computational techniques were combined with biophysical and spectroscopic methods to investigate the interaction between a selenoglycoside (SeDG) and galectins Gal-1 or Gal-3CRD. The integration of data from NMR, CD, and ITC provided valuable insights into designing selective inhibitors. The computational studies uncovered two different binding modes: when bound to Gal-1, SeDG adopted a V-shaped conformation driven by van der Waals interactions; on the contrary, when in complex with Gal-3CRD, it assumed an extended conformation. Comparing these modes identified specific interaction sites, guiding the design of selective inhibitors that can differentiate between the two galectins.

Noteworthy several computational studies have been conducted also for exploring protein-GAG interactions [175–176]. The study conducted by U. Uciechowska-Kaczmarzyk et al. [139] reports an extensive evaluation of protein–GAG complexes using a dataset of 28 complexes where the GAG length exceeded DP3 [139]. Through various statistical analyses to differentiate and highlight the docking programs with superior performance, valuable insights were provided into the most effective tools for studying these biologically relevant systems [177]. The interaction between the chemokine CXCL8/IL-8 and heparin-derived oligosaccharides was investigated by applying these docking procedures together with NMR spectroscopic techniques demonstrating the that higher affinity of the CXCL8 dimer for GAGs compared to the monomer and highlighting the structural plasticity that allows multiple binding modes. The use of HADDOCK in this context underscored its capability to model complex protein-GAG interactions accurately, providing a detailed understanding of the binding mechanisms at play [178].

## Conclusion

In structural biology, the investigation of protein–glycan interactions often relies on applying various structural techniques, including NMR, X-ray crystallography, and cryo-EM. Each of these methodologies comes with distinct advantages and limitations. NMR is particularly valuable for its ability to dynamically study molecules at the atomic level while preserving sample integrity. This makes it especially suitable for studying carbohydrates, offering insights into their 3D structures and conformations, but it generates a huge amount of data, which can be challenging to interpret effectively. X-ray crystallography provides high-resolution structural information, but unfortunately, this technique often fails when investigating carbohydrate–protein interactions due to the intrinsic flexibility of sugars, rendering them invisible in the density maps. In recent years, cryo-EM has seen widespread adoption in solving protein structures and glycoconjugates, thanks to significant advancements in instrumentation. Nevertheless, a notable limitation of cryo-EM lies in its capacity to handle large, intricate complexes. In this context, computational approaches can be valuable allies to develop accurate models helping in integrating and rationalizing data obtained from different methods and bridging the gap between the insights obtained from experimental data and the detailed understanding of complex biological systems. As example, models of protein and ligand, both in the free and bound states, can assist not only the interpretation of NMR spectra but also the building of structures that satisfy experimentally derived distance and angle restraints. Moreover, in X-ray crystallography and cryo-EM, protein models can be used to provide accurate templates for molecular replacement in the crystal cell or for backbone tracing and fitting sequence into a map, respectively.

We provided here an overview of computational tools available for ligand and protein building as well as the analysis of their molecular interactions, with a special focus on carbohydrates (Figure 5). Generally, to allow the prediction of an accurate 3D model of protein–glycan complexes, the combined use of different tools is highly recommended. A typical workflow could include firstly research to investigate the favoured carbohydrates bound to a protein (i.e., by using Glynet or LectinOracle), then other tools (such as CASPIF or PESTO) can be employed to predict the binding location. Subsequently, appropriate docking software (i.e., AutoDock Vina-Carb) can be used to provide a model of protein–glycan complex, which can be further refined (as example thanks to GlycanDock) and explored by molecular dynamic simulations (i.e., by using AMBER). Finally, the detailed analysis of the trajectory (i.e., by using AmberTools) provides unique vision of the 3D structure and real dynamics of glycan motifs in the bound state. Notably, the recent fusion of cutting-edge technologies, such as virtual reality, with interactive molecular simulations also allows to create an immersive environment, offering an opportunity without precedents to explore and manipulate molecular systems in real-time [179].

However, it is worth to note that, despite the continuous improvement of computational techniques and force fields development, there are still some limitations in the application of bioinformatic methods to the analysis of biomolecular interactions, especially in the case of complex carbohydrates, not to speak about bacterial glycans. Step forwards have been done in the improvement of docking programs dedicated to carbohydrates, however, the available software performed better for smaller glycans, while additional glycosidic linkages still remain a big challenge for docking calculations, and there is still room for improvement in ranking the best sugar docking pose. Additionally, over the years, different carbohydrate-specific force fields have been developed, the choice of which varies depending on the preferred simulation conditions, however, only few parameters have been defined for peculiar bacterial monosaccharides hampering a user-friendly and not time-consuming analysis of the system via MD simulations. A step change in this direction will permit the integrated use of valuable bioinformatic tools tailored on carbohydrates and would be of great help in unveiling critical structural and conformational features, at the atomic level, of complex glycans in the free and bound state, that can serve as essential resources for structural glycomics research to both experts and non-experts in glycobiology.

## Data Availability

Data sharing is not applicable as no new data was generated or analyzed in this study.
